# Supplementary material for: A fault-intrusion-tolerant system and deadline-aware algorithm for scheduling scientific workflow in the cloud
Source: PeerJ Comput Sci. 2021 Nov 2;7:e747. doi: 10.7717/peerj-cs.747 (PMC8576554; doi:10.7717/peerj-cs.747)
Supplement: Supplemental Information 6 [file peerj-cs-07-747-s006.zip › Myproject1.1/docs/org/cloudbus/cloudsim/power/package-use.html]

Uses of Package org.cloudbus.cloudsim.power


JavaScript is disabled on your browser.


- Overview
- Package
- Class
- Use
- Tree
- Deprecated
- Index
- Help

- Prev
- Next

- Frames
- No Frames

- All Classes

# Uses of Package org.cloudbus.cloudsim.power

- Packages that use org.cloudbus.cloudsim.power

  | Package | Description |
  | --- | --- |
  |  |  |
  | --- | --- |
  | org.cloudbus.cloudsim.examples.power |  |
  | org.cloudbus.cloudsim.power |  |
- Classes in org.cloudbus.cloudsim.power used by org.cloudbus.cloudsim.examples.power

  Class and Description

  PowerDatacenter

  PowerDatacenter is a class that enables simulation of power-aware data centers.

  PowerHost

  PowerHost class enables simulation of power-aware hosts.

  PowerVmAllocationPolicyMigrationAbstract

  The class of an abstract power-aware VM allocation policy that dynamically optimizes the VM
  allocation using migration.

  PowerVmSelectionPolicy

  The class of an abstract VM selection policy.
- Classes in org.cloudbus.cloudsim.power used by org.cloudbus.cloudsim.power

  Class and Description

  PowerDatacenter

  PowerDatacenter is a class that enables simulation of power-aware data centers.

  PowerHost

  PowerHost class enables simulation of power-aware hosts.

  PowerHostUtilizationHistory

  The class of a host that stores its CPU utilization history.

  PowerVm

  The class of a VM that stores its CPU utilization history.

  PowerVmAllocationPolicyAbstract

  The class of an abstract power-aware VM allocation policy.

  PowerVmAllocationPolicyMigrationAbstract

  The class of an abstract power-aware VM allocation policy that dynamically optimizes the VM
  allocation using migration.

  PowerVmAllocationPolicyMigrationLocalRegression

  The Local Regression (LR) VM allocation policy.

  PowerVmSelectionPolicy

  The class of an abstract VM selection policy.

- Overview
- Package
- Class
- Use
- Tree
- Deprecated
- Index
- Help

- Prev
- Next

- Frames
- No Frames

- All Classes
